# Cytoplasmic Skp2 Expression Is Associated with p-Akt1 and Predicts Poor Prognosis in Human Breast Carcinomas

**DOI:** 10.1371/journal.pone.0052675

**Published:** 2012-12-27

**Authors:** Jing Liu, Xiao-Long Wei, Wen-He Huang, Chun-Fa Chen, Jing-Wen Bai, Guo-Jun Zhang

**Affiliations:** 1 The Breast Center, The Affiliated Cancer Hospital of Shantou University Medical College, Shantou, Guangdong Province, PR China; 2 Cancer Research Center, Shantou University Medical College, Shantou, Guangdong Province, PR China; Health Canada, Canada

## Abstract

**Background:**

S-phase kinase protein 2 (Skp2), an oncogenic protein, is a key regulator in different cellular and molecular processes, through ubiquitin-proteasome degradation pathway. Increased levels of Skp2 are observed in various types of cancer and associated with poor prognosis. However, in human breast carcinomas, the underlying mechanism and prognostic significance of cytoplasmic Skp2 is still undefined.

**Methods:**

To investigate the role of cytoplasmic Skp2 expression in human breast carcinomas, we immnohistochemically assessed cytoplasmic Skp2, p-Akt1, and p27 expression in 251 patients with invasive ductal carcinomas of the breast. Association of cytoplasmic Skp2 expression with p-Akt1 and p27 was analyzed as well as correspondence with other clinicopathological parameters. Disease-free survival and overall survival were determined based on the Kaplan-Meier method and Cox regression models.

**Results:**

Cytoplasmic of Skp2 was detected in 165 out of 251 (65.7%) patients. Cytoplasmic Skp2 expression was associated with larger tumor size, more advanced histological grade, and positive HER2 expression. Increased cytoplasmic Skp2 expression correlated with p-Akt1 expression, with 54.2% (51/94) of low p-Akt1-expressing breast carcinomas, but 72.6% (114/157) of high p-Akt1-expressing breast carcinomas exhibiting cytoplasmic Skp2 expression. Elevated cytoplasmic Skp2 expression with low p-Akt1 expression was associated with poor disease-free and overall survival (DFS and OS), and Cox regression models demonstrated that cytoplasmic Skp2 expression was an independent prognostic marker for invasive breast carcinomas.

**Conclusion:**

Cytoplasmic Skp2 expression is associated with aggressive prognostic factors, such as larger tumor size, and advanced histological grade of the breast cancers. Results demonstrate that combined cytoplasmic Skp2 and p-Akt1 expression may be prognostic for patients with invasive breast carcinomas, and cytoplasmic Skp2 may serve as a potential therapeutic target.

## Introduction

Breast cancer is the most common malignancy in women. With breast cancer plaguing the United States as the second leading cause of cancer-related deaths amongst women, as well as increasing rates of cancer each year, there is a need to discover new prognostic markers and develop novel treatment strategies [1]. Histopathological classification divides breast carcinoma into several main types. Among them, invasive ductal carcinoma (IDC) is the most common type of breast cancer that displays aggressive clinical progression, as demonstrated by its rapid doubling time and early development of widespread metastasis.

Uncontrolled cellular proliferation, due to altered expression or activity of proteins involved in processes, such as cell cycle regulation, differentiation, and apoptosis, is the main hallmark of cancer [2]. The ubiquitination-proteasome system (UPS) plays a pivotal role in maintaining and regulating cellular homeostasis, and dysregulation of the UPS has emerged as a crucial player in cancer formation. S-phase kinase-associated protein 2 (Skp2) is an oncogenic member of the F-box family of proteins and constitutes the substrate recognition subunit of the Skp1-Cullin1-F-box protein (SCF) E3 ligase complex, substrates for Skp2 include the cyclin-dependent kinase inhibitor p27 and the activator of cyclin E, both of which interact with cyclin-dependent kinase 2 (CDK2) to regulate G1-S transition [3]. Skp2 has also been implicated in regulating the proteasome-mediated degradation of c-myc, p21, p57, and p130 [4].

Increased levels of Skp2 and reduced levels of p27 occur in various types of cancer, such as gastric carcinoma [5], prostate cancer [6], oral squamous cell carcinoma [7], and diffuse large B-cell lymphoma [8]. In breast cancer, Zheng *et al.* reported that high level of Skp2 expression were more frequently found in ER-negative tumors and tumors with metastatic axillary lymph nodes [9]. Traub *et al.* found that the combined assessment of Skp2 and p27 expression identifies aggressive breast cancer, and high Skp2 and low p27 expression indicates an unfavorable clinical course [10]. All the above mentioned studies analyzed the relationship of nuclear Skp2 expression with clinicopathological characteristics, concluding that nuclear Skp2 expression predicted a poor prognosis.

Recently, Gao *et al.* and Lin *et al.* both demonstrated that the activated, phosphorylated form of Akt1 (p-Akt1) interacts with and directly phosphorylates Skp2 to promote cytoplasmic localization of Skp2 and impair APC^Cdh1^-mediated Skp2 destruction [11], [12]. However, the biological significance of cytoplasmic Skp2 expression and its prognostic significance are still undefined in breast cancer. In this study, we evaluate subcellular Skp2 expression in invasive breast carcinoma by immunohistochemistry to analyze the relationship between p-Akt1 and cytoplasmic Skp2 expression, and correlate the presence of cytoplasmic Skp2 with patient survival.

## Materials and Methods

### Ethics Statement

The use of human tissues in this study was approved by the Academic Committee of Shantou University Medical College. This study was conducted according to the principles expressed in the Declaration of Helsinki.

### Patient information and reagents

All patients had undergone surgery at the Cancer Hospital of Shantou University Medical College between 1998 and 2008, and were without evidence of metastasis at the first visit. The mean patient age was 51±11 years (28–84 years old). All patients were diagnosed with invasive ductal carcinoma. The clinicopathological characters of the patients are summarized in Table 1.

**Table 1:** Relationship between low and high expression of cytoplasmic Skp2 with clinicopathological parameters.

		Skp2 cytoplasmic immunoreactivity					
Variables	No. of patients	Low	(%)	High	(%)	x^2^	*p* value
Age							
≤50 y	140	46	(32.9)	94	(67.1)	0.278	0.598
>50 y	111	40	(36.0)	71	(64.0)		
Menopausal status*							
Pre-	152	49	(32.2)	103	(67.8)	0.392	0.531
Post-	97	35	(36.1)	62	(63.9)		
Size of tumor							
≤2 cm	22	12	(54.5)	10	(45.5)	4.404	0.036
>2 cm	229	74	(32.3)	155	(67.7)		
AJCC stage							
I	11	6	(54.5)	5	(45.5)		
II	111	36	(32.4)	75	(67.6)	2.176	0.337
III	129	44	(34.1)	85	(65.9)		
Histological Grade							
1	26	15	(57.7)	11	(42.3)		
2	91	34	(37.4)	57	(62.6)	9.357	0.009
3	134	37	(27.6)	97	(72.4)		
Lymph node status							
Negative	91	30	(33.0)	61	(67.0)	0.106	0.744
Positive	160	56	(35.0)	104	(65.0)		
ER expression							
Negative	154	55	(35.7)	99	(64.3)	0.373	0.542
Positive	97	31	(32.0)	66	(68.0)		
PR expression							
Negative	170	57	(33.5)	113	(66.5)	0.126	0.723
Positive	81	29	(35.8)	52	(64.2)		
HER2 expression							
No	147	60	(40.8)	87	(59.2)	6.765	0.009
Yes	104	26	(25.0)	78	(75.0)		

* Two cases missing the information because of hysterectomy.

The antibodies used in this study were anti-phospho-Akt1 (Thr 308, sc-135650), anti-Skp2 (H-435, sc-7164), and anti-p27 (C-19, sc-528), all purchased from Santa Cruz Biotechnology (Santa Cruz, CA, USA).

### Immunohistochemistry

Formalin-fixed and paraffin-embedded tissues were cut into 4-micron-thick sections, stained with haematoxylin and eosin (H&E). Histological classification was made by two pathologists based on World Health Organization criteria and breast invasive ductal carcinomas were selected exclusively for analysis. Histological grade criteria was according to Scarff-Bloom-Richardson grade system [13].

Sections were deparaffinized in xylene and soaked in a series of graded alcohols for rehydration. Epitope retrieval was achieved by pretreatment with sodium citrate buffer, pH6.0, in a pressure cooker (for p-Akt1) or microwave (for Skp2 and p27). Slides were incubated at 4°C overnight with anti-p-Akt1 (dilution 1∶50), anti-Skp2 (dilution 1∶100), or anti-p27 (dilution 1∶150). In the negative controls, primary antibodies were omitted and replaced by PBS. 3% hydrogen peroxide was used for 30 minutes to inactivate endogenous peroxidase activity. Thereafter, sections were treated with peroxidase-conjugated goat anti-rabbit or anti-mouse antibodies. Counterstaining was carried out using haematoxylin.

**Figure 1:**
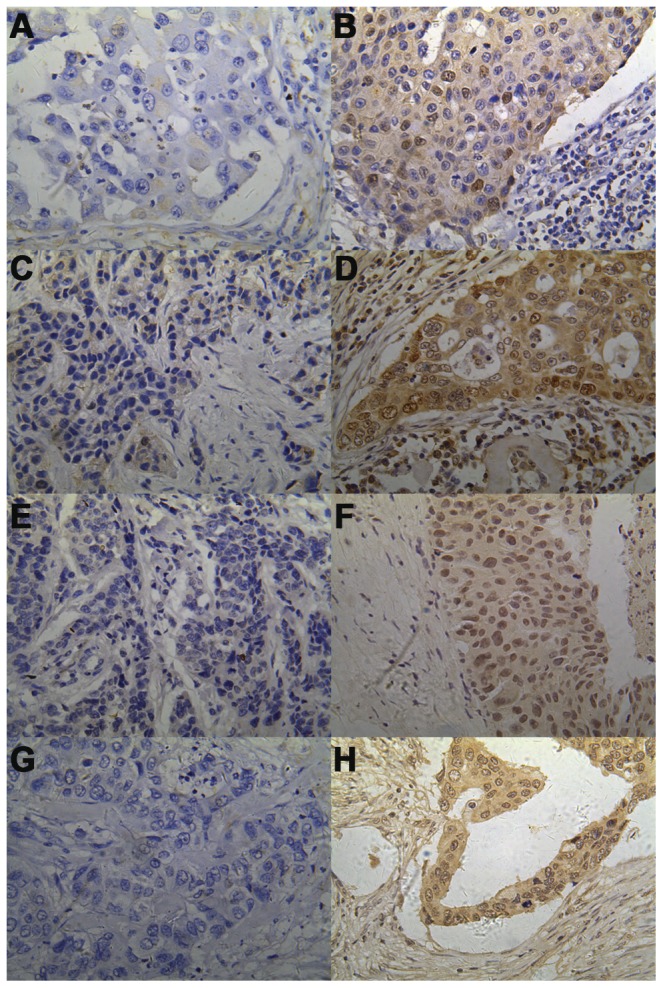
Immunohistochemical staining of Skp2, p-Akt1, and p27 in patients with invasive breast carcinoma. Representative images of immunohistochemical staining for Skp2, p-Akt1 and p27 in tissue of invasive breast carcinoma, A: low cytoplasmic Skp2 expression; B: high cytoplasmic Skp2 expression; C: low nuclear Skp2 expression; D: low nuclear Skp2 expression; E: low cytoplasmic p-Akt1 expression; F low cytoplasmic p-Akt1 expression; G: low p27 expression; and H: high p27 expression (Magnification: ×400).

Results were evaluated independently by two investigators with no prior knowledge of the patient data. Sections were visualized under a bright-field microscope (Olympus), and staining intensity and subcellular localization were evaluated twice in a blinded manner based on a pre-agreed staining scoring standard from specialized pathologists. For cytoplasmic p-Akt1 and Skp2 expression, no expression, weak expression, moderate expression, or strong expression was recorded as 0, 1, 2, and 3 for staining intensity, and the percentage of positive cells was also scored into 6 categories, 0 for <10%, 1 for 11–25%, 2 for 26–50%, 3 for 51–75%, 4 for 76–90%, and 5 for >90% [14], [15]. In the cases with a discrepancy between duplicated cores, the average score from the two tissue cores was taken as the final score. The level of Skp2 and p-Akt1 staining was calculated by adding up the scores of staining intensity and the percentage of positive cells to define low-expression (0–3) and high-expression (4–8). A positive staining of 50% of p27 cells was chosen as the cut-off point for discrimination of nuclear and cytoplasmic p27 expression, respectively (Figure 1).

### Follow-up and statistical analysis

Overall survival (OS) time was calculated in the months from the date of diagnosis and ended with the death of the patient or the last follow-up visit. The data on relapse and its date were used to calculate the disease-free survival (DFS), which was from the date of diagnosis and ended with the date of relapse. OS ranged between 4 and 138 months, with a mean ± SD of 61.7±32.6 months and a median of 62.8 months. At the end of the study, 74 breast cancer-specific deaths and 88 breast cancer relapses were observed, in which 22 patients occurred in the lung, 17 patients in the liver, 11 patients in the bone, 6 patients in the brain, 26 patients in mediastinum or distant lymph nodes, 15 patients recurred, 24 patients occurred in two or more organs and 32 patients occurred without location information. DFS ranged between 2–138 months, with a mean ± SD of 57.1±34.8 months and a median of 60.3 months.

Levels of statistical significance were evaluated with data by using the chi-square test or Fisher's exact test for categorical variables. The Spearman rank correlation coefficient was used to test the association between ordinal variables. Logistic regression was used to examine the association between cytoplasmic Skp2 expression and the various clinicopathological parameters. Survival curves were drawn according to the Kaplan-Meier method, and survival analysis was carried out using the log-rank test. Multivariate analysis, including hazard ratio using the Cox regression model, was done only on the variable with showing *p*<0.05 in the univariate analysis. All statistical differences were considered significant at the level of *p*<0.05. All data were analyzed with SPSS 16.0 for Windows.

## Results

### Clinicopathological analysis of cytoplasmic Skp2 expression in breast carcinoma

Clinicopathological analysis showed that the cytoplasmic Skp2 expression level (low vs. high) was not significantly associated with the age at diagnosis, menopausal status, lymph node metastasis, or AJCC stage (Table 1). In comparison with tumors less than 2 cm, tumors larger than 2 cm showed significant cytoplasmic Skp2 expression (x^2^ = 4.404, *p* = 0.036). Interestingly, high-expression of cytoplasmic Skp2 is more frequently observed in high-grade tumors (62.6% of grade 2 tumors and 72.4% of grade 3 tumors) than grade 1 tumors (42.3%) (x^2^ = 9.357 and *p* = 0.009). High cytoplasmic Skp2 high-expression was also associated with HER2 overexpression. In contrast, analysis of the clinicopathological significance of nuclear Skp2 expression in patients with breast carcinoma revealed no statistical significance between nuclear Skp2 expression and clinicopathological factors (Table 2).

**Table 2:** Relationship between low and high expression of nuclear Skp2 with clinicopathological parameters.

		Skp2 nuclear immunoreactivity					
Variables	No. of patients	Low	(%)	High	(%)	x^2^	*p* value
Age							
≤50 y	140	117	(83.6)	23	(16.4)	0.110	0.740
>50 y	111	91	(82.0)	20	(18.0)		
Menopausal status*							
Pre-	152	126	(82.9)	26	(17.1)	0.007	0.932
Post-	97	80	(82.5)	17	(17.5)		
Size of tumor							
≤2 cm	22	19	(86.4)	3	(13.6)	FE	0.459
>2 cm	229	189	(82.5)	40	(17.5)		
AJCC stage							
I	11	10	(90.9)	1	(9.1)		
II	111	96	(86.5)	15	(13.5)	2.836	0.242
III	129	102	(79.1)	27	(20.9)		
Histological Grade							
1	26	20	(76.9)	6	(23.1)		
2	91	78	(85.7)	13	(14.3)	1.224	0.542
3	134	110	(82.1)	24	(17.9)		
Lymph node status							
Negative	91	77	(84.6)	14	(15.4)	0.307	0.580
Positive	160	131	(81.9)	29	(18.1)		
ER expression							
Negative	154	127	(82.5)	27	(17.5)	0.045	0.832
Positive	97	81	(83.5)	16	(16.5)		
PR expression							
Negative	170	140	(82.4)	30	(17.6)	0.099	0.753
Positive	81	68	(84.0)	13	(16.0)		
HER2 expression							
No	147	126	(85.7)	21	(14.3)	2.024	0.155
Yes	104	82	(78.8)	22	(21.2)		

* Two cases missing the information because of hysterectomy.

### Variables associated with cytoplasmic Skp2 expression

In univariate logistic regression analyses, high cytoplasmic Skp2 expression was associated with large tumor size (B = 0.222, *p* = 0.036), high histological grade (B = 0.261, *p* = 0.008), and positive HER2 expression (B = 0.158, *p* = 0.009). In multivariable logistic regression analyses, the histological grade and HER2 expression remained statistically significant in the final model (each *p*<0.05).

### Relationship between expression of Skp2, p-Akt1 and p27 proteins

Spearman rank correlation showed a significant, positive linear correlation between the cytoplasmic expression of Skp2 and p-Akt1 (r = 0.187, *p* = 0.032), and a negative linear correlation between the cytoplasmic expression of Skp2 and p27, with r = −0.135 and *p* = 0.032 (Table 3). We also calculated the correlation between the cytoplasmic expression of p-Akt1 and cytoplasmic or nuclear p27 expression, both of which showed negatively correlated with statistical significance (Table 4).

**Table 3:** Relationship between the expressions of cytoplasmic Skp2 and p-Akt1 or p27 proteins.

	Skp2 cytoplasmic immunoreactivity				No. of patients	Spearman rank correlation	
	Low	(%)	High	(%)		r	P value
p27 cytoplasmic expression							
Low	64	(31.2)	141	(68.8)	205	−0.135	0.032
High	22	(47.8)	24	(52.2)	46		
p27 nuclear expression							
Low	67	(34.9)	125	(65.1)	192	0.024	0.704
High	19	(32.2)	40	(67.8)	59		
p-Akt1 cytoplasmic expression							
Low	43	(45.7)	51	(54.3)	94	0.187	0.003
High	43	(27.4)	114	(72.6)	157		

**Table 4:** Relationship between the expression of p-Akt1 and p27 proteins.

	p-Akt1 cytoplasmic immunoreactivity				No. of patients	Spearman rank correlation	
	Low	(%)	No.	(%)		r	P value
p27 nuclear expression							
Low	68	(33.2)	137	(66.8)	205	−0.187	0.003
High	26	(56.5)	20	(43.5)	46		
p27 nuclear expression							
Low	65	(33.9)	127	(66.1)	192	−0.134	0.034
High	29	(49.2)	30	(50.8)	59		

### Increased cytoplasmic expression of Skp2 is associated with poor survival of patients with breast carcinoma

**Figure 2:**
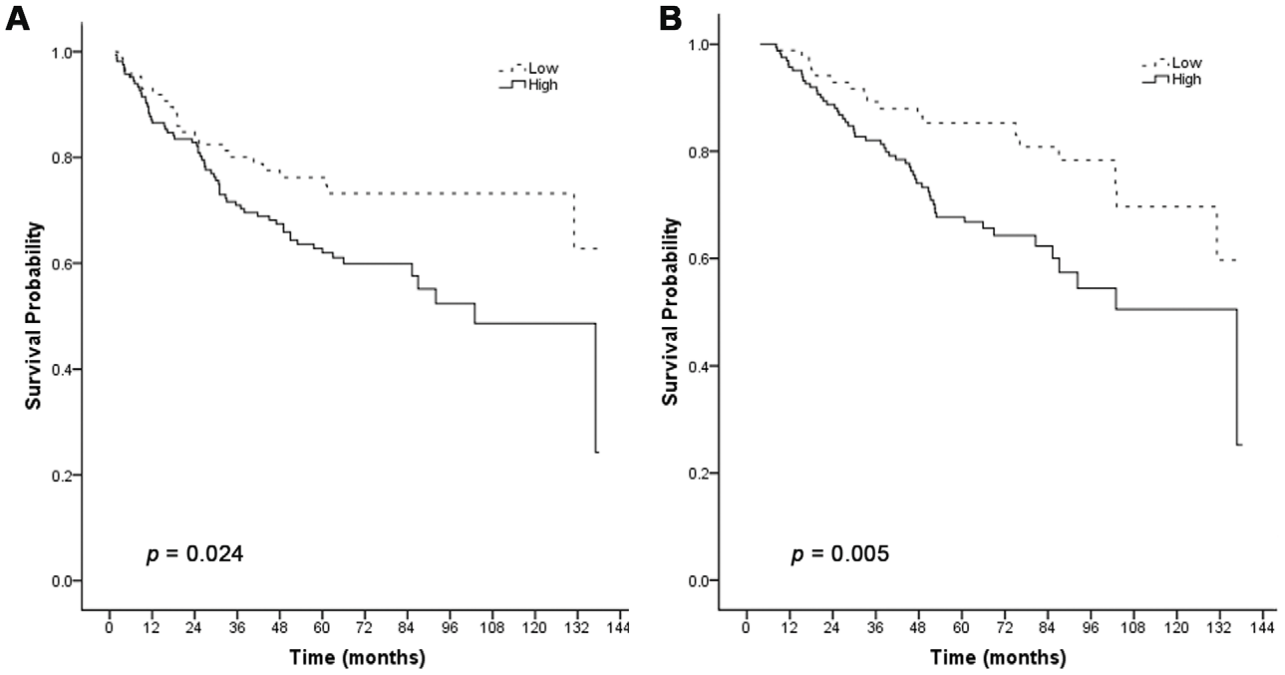
Cytoplasmic expression of Skp2 is associated with poor DFS and OS of patients with invasive breast carcinoma. Kaplan-Meier curves for the correlation between cytoplasmic Skp2 expression and DFS (A) or OS (B) in patients with invasive breast carcinoma.

The Kaplan-Meier curve and log-rank test analyses revealed that cytoplasmic Skp2 expression was significantly associated with DFS (log rank = 5.091, *p* = 0.024) (Figure 2A), and OS (log rank = 7.892, *p* = 0.005) (Figure 2B) in all breast invasive ductal carcinoma patients. Increased cytoplasmic Skp2 expression was associated with shorter DFS and OS, i.e. poorer prognosis of patients with breast invasive breast carcinoma.

### The correlation between cytoplasmic Skp2 expression and patient survival is affected by cytoplasmic p-Akt1 expression

**Figure 3:**
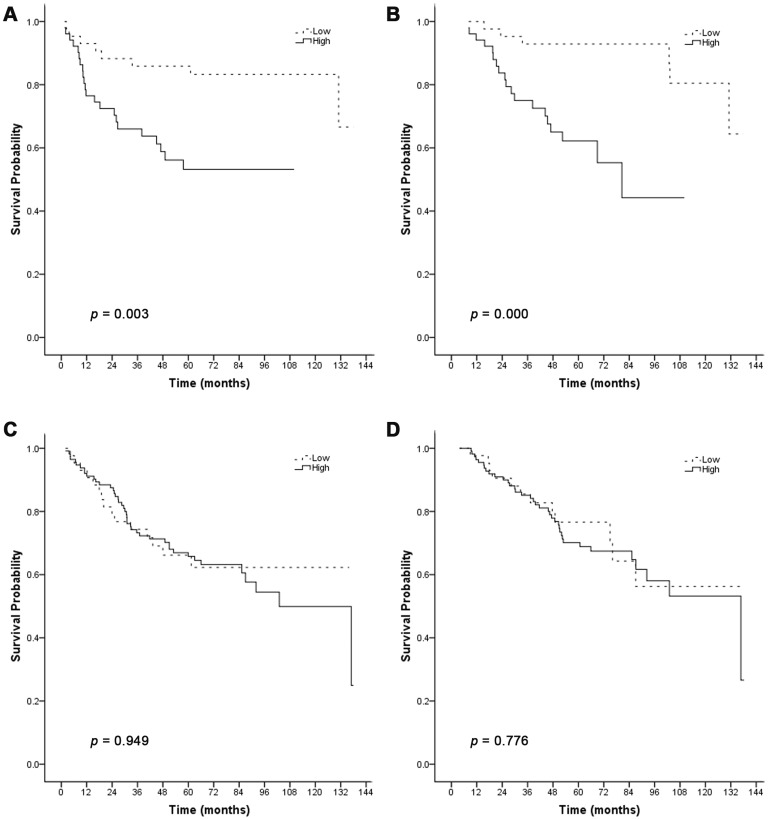
Relationship between DFS or OS and cytoplasmic Skp2 expression, stratified by cytoplasmic p-Akt1 expression. Kaplan-Meier curve analysis for the correlation between cytoplasmic Skp2 expression and DFS or OS in breast carcinoma patients with low cytoplasmic p-Akt1 expression (A and B), and high cytoplasmic p-Akt1 expression (C and D).

In order to investigate the effect of cytoplasmic p-Akt1 expression on the survival of invasive breast carcinoma patients with low or high cytoplasmic Skp2 expression, all patients with invasive breast carcinoma were categorized into low or high cytoplasmic p-Akt1 expression groups, and the Kaplan-Meier curve and log-rank test were applied to each group. We found that increased cytoplasmic expression of Skp2 was associated with poor DFS (log rank = 8.804, *p* = 0.003) (Figure 3A) and OS (log rank = 13.768, *p* = 0.000) (Figure 3B) in patients with low cytoplasmic p-Akt1 expression. However, in patients with high cytoplasmic p-Akt1 expression, cytoplasmic Skp2 expression did not affect either DFS (log rank = 0.004, *p* = 0.949) (Figure 3C) or OS (log rank = 0.081, *p* = 0.776) (Figure 3D).

### Cytoplasmic expression of Skp2 is an independent prognostic marker for invasive breast carcinoma

To determine the prognostic factors of invasive breast carcinomas, we applied a univariate Cox proportional hazards regression model to estimate the crude hazard ratios (HRs) of cytoplasmic Skp2 expression or clinicopathological variables on patient survival. We found that cytoplasmic Skp2 expression was significantly associated with DFS in breast carcinoma, confirming our Kaplan-Meier analysis. In addition to Skp2, cytoplasmic p27 expression, menopausal status, tumor size, histological grade, and lymph node status were also significantly associated with DFS in invasive breast carcinoma (Table 5). Cytoplasmic Skp2 expression, cytoplasmic p27 expression, tumor size, histological grade, and lymph node status were also significantly associated with OS (Table 6). Patients with high cytoplasmic Skp2 expression had poorer DFS (*p* = 0.024) with 1.731-fold (95% CI 1.068–2.774, *p* = 0.026) and OS (*p* = 0.005) with 2.119-fold (95% CI 1.240–3.621, *p* = 0.006) compared to those patients with low cytoplasmic Skp2 expression,

**Table 5:** Univariate and multivariate Cox proportional regression analysis on DFS of patients with invasive breast carcinoma.

Variables*	Univariate			Multivariate		
	HR	95%CI	P value	HR	95%CI	P value
Cytoplasmic Skp2 expression	1.731	1.068–2.774	0.026	1.662	1.027–2.690	0.039
Cytoplasmic p27 expression	0.530	0.288–0.977	0.042			
Nuclear p27 expression	0.675	0.392–1.161	0.155			
Cytoplasmic p-Akt1 expression	1.158	0.745–1.800	0.516			
Age 50 y	1.197	0.787–1.819	0.401			
Menopausal status*	1.543	1.012–2.350	0.044			
Tumor Sizeψ	1.162	1.069–1.264	0.000	1.164	1.063–1.274	0.001
Histological Grade§	4.600	1.432–14.774	0.010			
Lymph node status	3.677	2.075–6.516	0.000	3.835	2.158–6.813	0.000

* Two cases missing the information because of hysterectomy.

ψ Tumor size was calculated as continuous variable.

§ Histological grade was compared between grade1 and grade 2 & 3.

**Table 6:** Univariate and multivariate Cox proportional regression analysis on OS of patients with invasive breast carcinoma.

Variables*	Univariate			Multivariate		
	HR	95%CI	P value	HR	95%CI	P value
Cytoplasmic Skp2 expression	2.119	1.240–3.621	0.006	2.084	1.217–3.568	0.007
Cytoplasmic p27 expression	0.500	0.256–0.976	0.042			
Nuclear p27 expression	0.563	0.303–1.045	0.069			
Cytoplasmic p-Akt1 expression	1.293	0.798–2.097	0.297			
Age 50 y	1.224	0.774–1.933	0.387			
Menopausal statusθ	1.349	0.851–2.139	0.203			
Tumor Sizeψ	1.166	1.067–1.274	0.001	1.166	1.061–1.283	0.002
Histological Grade§	4.125	1.276–13.336	0.018			
Lymph node status	3.399	1.830–6.313	0.000	3.542	1.901–6.598	0.000

* 3 cases are censored cases before the earliest event in a stratum.

θ Two cases missing the information because of hysterectomy.

ψ Tumor size was calculated as continuous variable.

§ Histological grade was compared between grade1 and grade 2 & 3.

To identify the independent prognostic factors for invasive breast carcinoma, we next used multivariate Cox proportional hazard analysis to analyze patient survival, and only variables with *p*<0.05 in the univariate analysis were included in the regression model (Tables 5 & 6). Multivariate analysis showed that cytoplasmic Skp2 expression (HR = 1.662, 95% CI 1.027–2.690, *p* = 0.039) parallels prognostic indicators of metastasis, as do tumor size (HR = 1.164, 95% CI 1.063–1.274, *p* = 0.001) and lymph node status (HR = 3.835, 95% CI 2.158–6.813, *p* = 0.000). Furthermore, cytoplasmic Skp2 expression (HR = 2.084, 95% CI 1.217–3.568, *p* = 0.007) also parallels prognostic indicators of survival, as well as tumor size (HR = 1.166, 95% CI 1.061–1.283, *p* = 0.002) and lymph node status (HR = 3.542, 95% CI 1.901–6.598, *p* = 0.000). These data indicate that cytoplasmic Skp2 expression is an independent prognostic factor for recurrence/metastasis and survival of patients with breast carcinoma.

## Discussion

Many molecular markers have been demonstrated with prognostic value. However, few of them have been evaluated as predictive markers offering the chance for specific molecular-targeted therapies and the data available mostly seem to be inconclusive or at least controversial partly [16].

Skp2, as an oncogenic protein, plays a pivotal role in various types of cancers. However, the role of cytoplasmic expression of Skp2 in cancers remains undefined. In the present study, we immunohistochemically determined the subcellular expression of Skp2 in invasive ductal carcinomas of the breast and studied its clinicopathological significance. Our data showed that elevated cytoplasmic expression of Skp2 correlated significantly with larger tumor size, advanced histological grade, and positive HER2 expression, suggesting that overexpression of cytoplasmic Skp2 could be associated with fast proliferation, aggressive cellular behavior and potentially poor prognosis for patients with invasive ductal breast cancers.. Cytoplasmic Skp2 protein expression showed a significant correlation with p-Akt1. These results have shown for the first time that cytoplasmic Skp2 is overexpressed with p-Akt1 in invasive breast carcinomas.

Previous *in vitro* studies demonstrate that Skp2 can be phosphorylated by activated Akt1 [11], [12]. We extend these results to show that a significant correlation exists between cytoplasmic Skp2 and p-Akt1 expression in patients, and suggests that increased cytoplasmic Skp2 expression may be at least partly due to Akt1 activation in invasive breast carcinomas. Cytoplasmic relocalization of Skp2 expression is associated with rapid proliferation, aggressive cellular behavior, and potentially with poor prognosis for breast carcinoma patients. As expected, patients with cytoplasmic Skp2 expression showed significantly poorer survival for both DFS and OS in univariate and multivariate analyses. Lin *et al.* reported that cytosolic Skp2 mediates cell migration, suggesting that cytosolic Skp2 may play an important role in tumor invasion and metastasis [12]. Our results demonstrate that cytoplasmic Skp2 may facilitate not only progression, but also metastasis in breast carcinoma patients.

**Figure 4:**
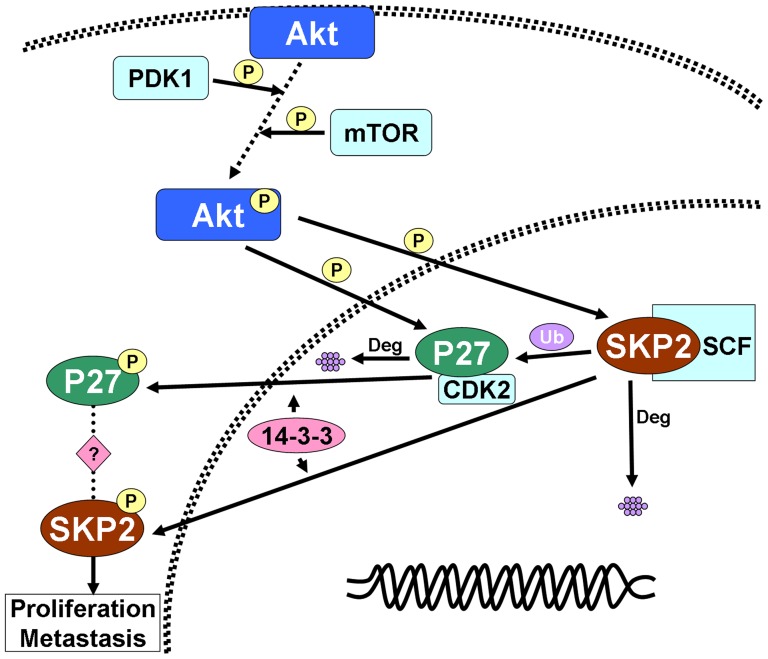
Schematic diagram of the Akt-Skp2 pathway. Activation of Akt by PDK1 and mTOR results in phosphorylation of Skp2 and p27 protein, which are then transported to the cytoplasm with exportin 14-3-3, avoiding degradation. Our results suggest that cytoplasmic Skp2 may facilitate proliferation and metastasis of breast carcinoma, although the function and relationship between cytoplasmic Skp2 and p27 in breast carcinomas remains unclear.

Interestingly, in patients with low p-Akt1 expression, cytoplasmic Skp2 expression was significantly associated with DFS and OS of patients with breast carcinoma. In contrast, in patients with high cytoplasmic expression of p-Akt1, Skp2 levels did not influence DFS or OS. Akt1 phosphorylates many downstream proteins related to cell cycle regulation, cell proliferation, and UPS, and trigger oncogenic signaling. Similar to Skp2, Akt1-mediated phosphorylation of the CDK inhibitor p27 causes translocation of p27 to the cytoplasm. The cytoplasmic relocalization of phosphorylated p27 relieves CDK2 from p27-mediated inhibition, thus resulting in cell cycling and tumor-cell proliferation [17]. Recently, Chan *et al.* reported that a distinct E3 ligase, SCF-Skp2, is utilized by diverse growth factors to regulate Akt ubiquitination, Herceptin sensitivity and tumorigenesis in Her2-positive tumors [18]. Another study pointed that atypical protein kinase C (PKC) promotes metastasis and enhanced cell resistance to anoikis via the PKC-SKP2-AKT pathway [19]. So there might be crosstalk between Skp2 and Akt1, through different regulatory mechanism. And in the patients with high p-Akt1 expression, activated Akt1 may phosphorylate both Skp2 and p27, and transfer them to cytoplasm. There may be other mechanism to phosphorylation and mislocalization of Skp2 to cytoplasm, such as CDK2 and Cdc14B [20]. In the absence of activated Akt1, whether and how cytoplasmic Skp2 is involved in the development of metastasis and cancer progression remains to be elucidated (Figure 4).

A splice variant of Skp2, i.e. Skp2B with a variant C-terminal, has been reported to play different roles in breast cancers [21], [22], [23]. The antibody used in the present study is supposed to recognize both full-length human Skp2 and Skp2B variant. In this study, we could not differentiate the cytoplasmic full-length Skp2 from Skp2B. Nevertheless, it is clear that the cytoplasmic expression of Skp2, as evidenced by immunohistochemistry, is indicative of aggressive biological behavior and poorer prognosis. Whether the variant, Skp2B will be phosphorylated by p-Akt1 and affect biological function of breast cancer cells needs to be further studied.

The pattern of Skp2 expression in breast cancers, as evidenced by immunohistochemistry, is highly heterogeneous. Our results demonstrated that combined cytoplasmic Skp2 and p-Akt1 expression serve as a prognostic marker. For invasive breast carcinoma patients with high cytoplasmic Skp2 and low p-Akt1 level, Skp2 inhibitors may represent a potential therapeutic strategy.
