# Correction: Cytoplasmic Skp2 Expression Is Associated with p-Akt1 and Predicts Poor Prognosis in Human Breast Carcinomas

**DOI:** 10.1371/annotation/d8502df1-611d-4d4a-b697-acaee15ac8c8

**Published:** 2013-07-15

**Authors:** Jing Liu, Xiao-Long Wei, Wen-He Huang, Chun-Fa Chen, Jing-Wen Bai, Guo-Jun Zhang

A second grant from the National Natural Science Foundation of China is missing from the Funding section. The second grant number is 31271068. 

